# Core Measures for Congestive Heart Failure in a Tertiary Care Setting in Pakistan

**DOI:** 10.7759/cureus.728

**Published:** 2016-08-08

**Authors:** Rizwan Zafar, Muhammad Haris, Salman Assad, Muhammad Usman Shabbir, Haider Ghazanfar, Sarah A Malik, Tehreem Khalid, Ali H Abbas, Asad A Saleem

**Affiliations:** 1 Department of Cardiology, Shifa College of Medicine, Islamabad, Pakistan; 2 Department of Neurology & Neurosurgery, Shifa Tameer-e-Millat University, Islamabad, Pakistan; 3 Department of Urology, Shifa College of Medicine, Islamabad, Pakistan; 4 Department of Pathology, Shifa College Of Medicine, Islamabad, Pakistan; 5 Department of Internal Medicine, Shifa College of Medicine, Islamabad, Pakistan; 6 Shifa College of Medicine, Islamabad, Pakistan

**Keywords:** heart failure, morbidity, mortality, pakistan, chronic disease management

## Abstract

Purpose: Heart failure presents a huge burden for individual patients and the healthcare system as a whole. This study aims to assess the adherence to these core measures as identified by the Joint Commission on Accreditation of Healthcare Organizations (JCAHO)/ American Heart Association (AHA) by physicians of Pakistan.

Materials and Methodology: We conducted a cross-sectional study in Shifa International Hospital, Islamabad, Pakistan from the period of April 2013 to April 2016. Patients with a primary diagnosis of heart failure were drawn from a coding section of hospital’s record department. Data was evaluated to assess how strictly doctors were following core measures identified by JCAHO/AHA for the given diagnosis. Inclusion criteria for this study were patients ≥ 17 years of age and patients with a primary diagnosis of heart failure according to New York Heart Association (NYHA) classification. Patients with congenital anomalies and structural heart wall problems, like sarcoidosis, hemochromatosis, and amyloidosis, were excluded from the study.

Results: Mean ejection fraction (EF) was found to be 27.23 ± 11.72 percent. Symptoms assessment of heart failure was done in 16/421 (3.8%) patients according to NYHA classification and in 405/421 (96.2%) patients according to outpatient-based heart failure assessment based on physician's experience other than NYHA classification. Left ventricle ejection fraction (LVEF) was assessed in 411/421 (97%) patients. Out of these, 336/411 (81.7%) patients had EF < 40%. Mean EF was found to be significantly higher in females as compared to males (p < 0.001). Three hundred and thirty-six out of 411 (81.7%) patients with EF < 40% needed angiotensin converting enzyme inhibitors (ACEi) and beta-blocker (BB) prescriptions. ACEi were prescribed only to 230/336 (68.7%) patients and 248/336 (73.8%) patients were given BB with documented contraindication to ACEi and BB in 7.36% and 17% patients, respectively. There was no significant association between gender and mean duration of hospitalization (p = 0.411). No significant association was found between EF ≤ 40% and mean duration of hospitalization (p = 0.426).

Conclusion: We found that symptom assessment of congestive heart failure (CHF) patients, according to NYHA guidelines, are strikingly low. Also, a significant percentage of patients who need ACEi and BB are not prescribed the required medications despite echocardiography showing low left ventricular function.

## Introduction

Heart failure presents as a huge burden for individual patients and the healthcare system as a whole. There are an estimated 23 million people suffering from heart failure (HF) worldwide with a prevalence of 1-3%. There is a 10% increase in heart failure prevalence above 65 years of age with a five-year mortality rate of up to 75% following the first hospital admission [1-2]. Despite therapeutic advancements, the prevalence of heart failure is increasing day by day, which results in great capital expenditures, deterioration of the quality of life, and mortality. The situation in Pakistan is no better as a study shows increasing numbers as well as increasing hospitalizations due to heart failure [3]. In Pakistan, no national census guidelines exist for the management of HF. Physician tends to follow already established guidelines from the American Heart Association (AHA/JCAHO) or European Cardiologists Society (ECS). Little is known about the adherence to these standard-of-care measures in tertiary care settings in Pakistan. It has been well established that adherence to these guidelines improves outcomes in CHF patients [4-5]. This study aims to assess adherence to these core measures identified by the Joint Commission on Accreditation of Healthcare Organization (JCAHO) by physicians in Pakistan [6-8].

## Materials and methods

We conducted a cross-sectional study of the patients previously treated at the Shifa International Hospital, Islamabad, Pakistan. After approval by the Institutional Review Board (IRB) of Shifa International Hospital, lists of patients discharged from cardiology and medicine wards from the period of April 2013 to April 2016 with a primary diagnosis of HF were drawn from a coding section of hospital’s record department. Files were retrieved and reviewed; those meeting the eligibility criteria were included in the study. Data was evaluated to assess how strictly doctors were following core measures identified by the JCAHO/AHA for the given diagnosis. The study population included adult patients with a diagnosis of heart failure. Inclusion criteria for this study were patients ≥ 17 years of age and patients with a primary diagnosis of heart failure according to NYHA classification. Patients with congenital anomalies and structural heart wall problems, like sarcoidosis, hemochromatosis, and amyloidosis, were excluded from the study. 

The primary outcome was to evaluate overall hospital adherence to each and every core measure recommended by the JCAHO (Table 1).

**Table 1:** Core Measures by Joint Commission on Accreditation of Healthcare Organizations (JCAHO) ACE = angiotensin converting enzyme; EF = ejection fraction

Core Measures - JCAHO
HF-1: Assessment of the patient's symptoms according to NYHA classification.
HF-2: Evaluation and documentation of the left ventricular function of the patient (within last 12 months).
HF-3: Prescription of ACE inhibitors if EF < 40%. (If there is any contraindication to ACE inhibitors and beta-blockers on medical grounds, then it should be documented.)
HF-4: Prescription of beta-blockers if EF < 40%. (If there is any contraindication to ACE inhibitors and beta-blockers on medical grounds, then it should be documented.)
HF-5: Patient education regarding activity, diet control, drug compliance, and follow-up.

Data were analyzed using SPSS version 21. Independent sample t-test was applied to assess whether there was any significant difference between the mean age of female and male participants. The Mann-Whitney test was used to assess for any association of gender with mean ejection fraction and mean duration of hospitalization.

## Results

Total admissions in cardiology and medicine wards with a primary diagnosis of heart failure (HF) were 654 out of which 420 were eligible for the study. The mean age of the participants was found to be 66 ± 12.5 years. The mean age of male participants was 65.82 ± 12.96 years while the mean age of female participants was 66.53 ± 11.77 years. Independent sample t-test was applied to assess whether there was any significant difference between the mean age of female and male participants. No significant difference was found between the groups (p > 0.05). Out of these, 218/420 (51.9%) were males and 202/420 (48.1%) were female patients. Echocardiography and left ventricular ejection fraction (LVEF) assessment were done for diagnosis in 411/420 (97.9%) patients. Mean ejection fraction (EF) was found to be 27.23 ± 11.72 percent. Symptoms assessment of heart failure was done in 16/421 (3.8%) patients according to NYHA classification and in 405/421 (96.2%) patients according to outpatient-based heart failure assessment based on physician's experience other than NYHA classification. 

Out of a total of 411 patients, 336 (81.7%) patients had EF < 40% and needed ACEi and BB prescriptions. Out of these 336 potential candidates, 179 (53.27%) were given both ACEi and BB, 51 (15.17%) patients were only given ACEi, and 69 (20.53%) patients were only given BB. Out of 190 patients who were not prescribed ACEi, contraindication was documented in 14 (7.36%) patients. Out of 88 patients who were not prescribed beta-blockers, contraindication was documented in 15 (17%) patients. Four hundred and one (98.5%) patients were given counseling and educated regarding diet, activity, and follow-up. The mean duration of hospitalization was 4.83 ± 3.42 days (Table 2).

**Table 2:** Prescription of ACE Inhibitors and B-Blockers ACE = angiotensin converting enzymes; EF = ejection fraction; B-blockers = beta blockers

Variables	Frequency & Percentage
Echocardiography done	411/420 (97.8%)
EF < 40%	336/411 (81.7%)
ACE inhibitors prescribed	230/336 (68.4%)
ACE inhibitors contraindicated	14/336 (0.04%)
B-blockers prescribed	248/336 (73.8%)
B-blockers contraindicated	15/336 (0.04%)

**Figure 1:**
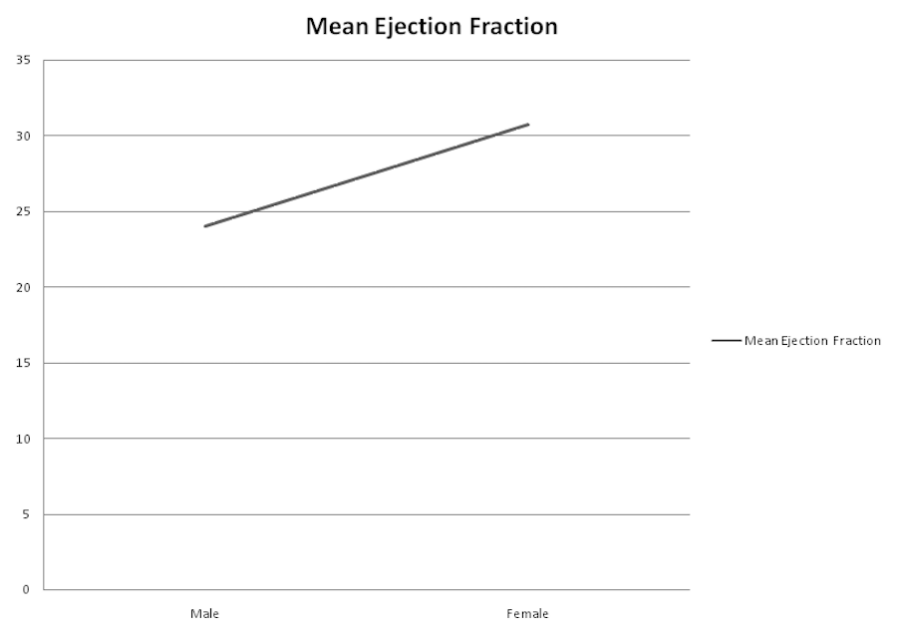
Association of Mean Ejection Fraction with Gender

The Mann-Whitney test was used to assess for any association of gender with mean ejection fraction and mean duration of hospitalization. Mean ejection fraction was found to be significantly higher in females as compared to males (p < 0.001). There was no significant association between gender and mean duration of hospitalization (p = 0.411). No significant association was found between EF ≤ 40% and mean duration of hospitalization (p = 0.426) (Table 3, Figure 1).

**Table 3:** Association of Gender with Mean Ejection Fraction and Hospital Stay

	Male	Female	P-value
Mean Ejection Fraction	24.0 ± 9.924	30.71 ± 12.51	< 0.001
Mean Duration of Stay	5.01 ± 3.55	4.64 ± 3.28	< 0.411

## Discussion

This survey showed variability in adherence to each core measure for congestive heart failure. Compliance with the assessment of symptoms according to NYHA classification was minimum probably because physicians tend to rate the symptoms by their own scoring system. Physicians should be more objective in assessing the symptoms according to the well-developed NYHA classification, so that at following visits they can use that categorization as a benchmark to see if patients are worsening, improving, or are at the same level. LVEF was assessed in the majority of the patients who were diagnosed with congestive failure. Unfortunately, a significant portion of the patients was not given ACEi and BB despite clear indication. Written discharge instructions show good adherence. Our findings are consistent with studies conducted in Western countries as poor adherence to these evidence-based measures has also been reported in the Western studies where these guidelines were formulated [9-12]. The South Asian population has a greater prevalence of coronary artery disease (CAD), which is also a leading cause of heart failure [13-14]. This fact itself emphasizes the necessity for the development of proper management planning and guidelines according to Asian population genetics, which, unfortunately, do not exist thus far. That is why it is ideal to follow AHA or European Society of Cardiology (ESC) guidelines. Although it is not known whether these guidelines are helpful in alleviating the morbidity and mortality rate in the Pakistani population, it is well established in Western countries [7-8].

Males were more likely to have decreased mean ejection fraction as compared to females. Our results were similar to a study done on Italian population, which concluded that Italian men were more likely to have reduced ejection fraction as compared to Italian women (1.59% versus 1.5%) [15]. According to a large multicenter study based on reduced or preserved EF heart failure, women, when compared to men, were found to be older and more likely to have hypertension, depression, or valvular heart disease and less likely to have coronary artery disease or peripheral vascular disease [16]. Valsartan in an acute myocardial infarction (MI) trial concluded that female patients with EF < 40% who were discharged after being treated for MI were more likely to be admitted for HF during follow-up as compared to male patients [17]. A meta-analysis concluded that patients who had heart failure with preserved ejection fraction were at a lower risk of death than the patients who had heart failure with reduced ejection fraction regardless of age, gender, and etiology of cardiac failure [17]. Studies have shown that usage of ACEi is associated with improved outcomes in patients with heart failure and reduced ejection fraction [18]. The same results have not been seen in patients who have heart failure but have a preserved ejection fraction. According to the PEP-CHF randomized controlled trial, ACEi were found to have no effect on the mortality of heart failure with preserved ejection fraction [19]. A review concluded that the median prevalence of use of ACE inhibitors in patients with heart failure and reduced ejection fraction was found to be 71% in patients discharged from hospitals and 86% in patients discharged from specialty clinics [20]. In a study done in Austria by Bungard, et al., they reported that 65% of the patients with HF and reduced EF were discharged on ACE inhibitors [21]. In our study, ACE inhibitors were prescribed to 230/336 (68.7%) patients with reduced ejection fraction. Beta-blockers are one of the mainstay drugs in treating patients with heart failure and reduced ejection fraction [22]. These have been shown to significantly decrease mortality in patients with heart failure with reduced ejection fraction, most likely due to reduced detrimental effects of catecholamine stimulation on the myocardium [23]. According to the Organized Program to Initiate Lifesaving Treatment in Hospitalized HF Patients (OPTIMIZE-HF) study, the prescription rate of beta blockers at discharge was increased from 76.3% to 86.4% during the course of study while no change in the ACE inhibitor prescription rate was seen [24]. Beta-blockers were prescribed to 73.8% of the patients in our study, which is higher than the 68.7% prescription rate of ACE inhibitors.

Lack of adherence to these guidelines may be attributed to a lack of knowledge about such guidelines by Pakistan physicians as well as a failure to put this knowledge into practice. This has been depicted by a Pakistan-based study, which showed that only 92.1% of cardiologists practicing in major cities of Pakistan were aware of current guidelines of the AHA for CHF and only 87.2% said that they follow these guidelines closely [12]. Another factor that might be contributing to the poor adherence is the economic condition of the patients. Because government spending on health is very low, patients have to bear the medication cost for themselves so sometimes physicians are forced to underprescribe appropriate medications. Poor patient compliance might contribute to inadequate therapy administration [25]. According to the IMPACT-RECO survey, underprescribing of ACEi and BB was significantly associated with patients with age > 75 [25].

### Strengths and weaknesses

In Pakistan, no such data is available regarding adherence to these qualities of core measures and no clinical audit has been ever conducted on a large scale. On the other hand, it is observed in a US-based study that adherence to these measures improved over time by conducting the clinical audit in the hospital [11]. The results of this single-center tertiary care hospital-based study do not depict the overall management provided to CHF patients across Pakistan. The drawbacks of our analysis also include non-documentation of patients' compliance with dietary instructions. Also, the physicians of the institute from where the data has been obtained are mostly United States/United Kingdom-trained so they tend to follow these guidelines more often than those who are trained elsewhere in the world.

### Suggestions

1. In order to decrease the morbidity and mortality in CHF patients, an awareness program should be started to ensure that these guidelines are being followed.

2. To evaluate the usefulness of these guidelines in a Pakistani population, there is a great need of a prospective study in a group of patients who are managed according to guidelines of AHA/JCAHO or the ECS guidelines. It would really help in convincing physicians to recognize and adhere to globally accepted criteria for management of CHF patients.

3. An annual audit of physicians should be conducted by hospitals to let physicians know how well they are performing in accordance with the guidelines.

## Conclusions

We found that symptom assessment of CHF patients according to NYHA guidelines are strikingly low and also a significant percentage of CHF patients with reduced EF are not being prescribed the required ACE inhibitors and beta blockers.
